# Clinical Decision Support for Chronic Kidney Disease in Primary Care: A Cluster Randomized Clinical Trial

**DOI:** 10.1001/jamanetworkopen.2026.11112

**Published:** 2026-05-08

**Authors:** Xizi Zheng, Miao Hui, Hongyu Yang, Zhao Yang, Linger Tang, Youlu Zhao, Lingyi Xu, Qingqing Zhou, Jingwei Wang, Mengrui Li, Shuhong Zhu, Fengjuan Gao, Jing Li, Jicheng Lv, Li Yang

**Affiliations:** 1 Renal Division, Peking University First Hospital, Institute of Nephrology, Peking University, Key Laboratory of Renal Disease, Ministry of Health of China, Beijing, China; 2 Beijing Key Laboratory of Precision Medicine and New-drug/Equipment Development for Severe Kidney Disease, Beijing, China; 3 Key Laboratory of CKD Prevention and Treatment, Ministry of Education of China, Beijing, China; 4 Research Units of Diagnosis and Treatment of Immune-Mediated Kidney Diseases, Chinese Academy of Medical Sciences, Beijing, China; 5 Division of Sleep Medicine, Peking University People’s Hospital, Beijing, China; 6 Office of Discipline and Talent, Peking University First Hospital, Beijing, China; 7 Xicheng District Center for Smart Health and Digital Intelligence, Beijing, China; 8 Beijing Xicheng District Desheng Community Health Service Center, Beijing, China

## Abstract

**Importance:**

Optimal clinical decision support system (CDSS) implementation for chronic kidney disease (CKD) management in Chinese primary care remains undefined despite the high disease burden.

**Objective:**

To examine whether a CDSS for CKD could improve physician behavior and patient outcomes in primary care.

**Design, Setting, and Participants:**

This cluster randomized clinical trial is being conducted in Chinese primary care centers. The trial spans a 3-year period (January 1, 2023, to December 31, 2026) and is divided into 2 phases; this phase 1 analysis includes data from the initial 6-month follow-up (June 10 to December 10, 2023). Centers were stratified by size and randomized 1:1 to intervention or control. Participants are adults (aged ≥18 years) with CKD who had 2 visits or more during the 1-year screening period, all enrolled before randomization.

**Interventions:**

Both groups received government-supported, nephrologist-delivered training on CKD management. The intervention group was additionally equipped with a CDSS embedded into the electronic health record.

**Main Outcomes and Measures:**

The primary outcome was a 36-month composite of kidney-related and cardiovascular hospitalizations (phase 2). This phase 1 analysis evaluated 6-month process measures (ie, CKD diagnosis and renin-angiotensin-aldosterone system inhibitor or sodium-dependent glucose transporter 2 inhibitor use) and clinical outcomes (ie, blood pressure, glycated hemoglobin, and low-density lipoprotein cholesterol control).

**Results:**

A total of 3390 patients (mean [SD] age, 72.0 [10.2] years; 1881 [55.5%] female) from 30 primary care centers were included (1912 in the intervention group and 1478 in the control group). Follow-up at 6 months was completed by 3055 patients (90.1%; 1743 [91.2%] in the intervention group and 1312 [88.8%] in the control group). CKD diagnosis rates increased by 21.4 (95% CI, 18.6-24.3) percentage points in the intervention group and by 27.9 (95% CI, 24.4-31.3) percentage points in the control group, with a nonsignificant between-group difference (adjusted odds ratio [AOR], 0.91; 95% CI, 0.72-1.14). Renin-angiotensin-aldosterone system inhibitor use (AOR, 0.96; 95% CI, 0.78-1.19), sodium-dependent glucose transporter 2 inhibitor use (AOR, 1.02; 95% CI, 0.78-1.32), and low-density lipoprotein cholesterol control (AOR, 1.10; 95% CI, 0.83-1.46) showed parallel improvements with no between-group differences. Blood pressure (AOR, 0.88; 95% CI, 0.71-1.09) and glycated hemoglobin (AOR, 1.22; 95% CI, 0.74-2.01) control showed no improvement.

**Conclusions and Relevance:**

In this cluster randomized trial of a CDSS for CKD in primary care, both the intervention and control groups demonstrated comparable improvements in 6-month outcomes, with no independent effect of the CDSS detected.

**Trial Registration:**

Chinese Clinical Trial Registry Identifier: ChiCTR2300070555

## Key Points

**Question:**

Does a clinical decision support system (CDSS) improve chronic kidney disease management in a primary care system with high disease burden and limited resources?

**Findings:**

In this cluster randomized trial of 3390 patients with chronic kidney disease from 30 primary care centers in China, both the intervention and control groups demonstrated comparable improvements over 6 months, with no significant additional benefit from a CDSS.

**Meaning:**

These findings suggest that in resource-limited primary care, targeted education and policy support may be more foundational, whereas a CDSS should focus on tiered interruptive alerts with feedback-driven adjustments.

## Teaser

This cluster randomized clinical trial of patients with chronic kidney disease assesses the effectiveness of a clinical decision support system in improving process measures and clinical outcomes in primary care.

## Introduction

Chronic kidney disease (CKD) poses an escalating global health burden, particularly in low- and middle-income countries.^1^ China has an estimated 120 million CKD cases, predominantly early-stage, yet most patients presenting in primary care remain underdiagnosed and inadequately treated.^2^ Contributing factors include limited awareness among frontline primary care physicians (PCPs)^3^ and challenges in implementing guidelines.^4^

Digital tools for CKD management have evolved. Basic kidney function reporting improved kidney referral rates^5^ but did not increase use of key therapies, such as renin-angiotensin-aldosterone system inhibitors (RAASis).^6,7^ More comprehensive clinical decision support systems (CDSSs) have yielded only modest improvements.^8,9,10,11^ This limited effectiveness may reflect a high baseline care standard in high-income countries where these systems have been evaluated. For example, a Canadian study^7^ found that more than 75% of eligible patients with CKD already received RAASis at baseline, leaving little room for improvement.

China’s CKD burden, weak primary care system, and limited nephrology resources^2^ create a compelling setting for a CDSS. However, no trials have evaluated such interventions in Chinese primary care. We therefore developed a multidisciplinary CDSS for CKD and conducted a multicenter cluster randomized trial to assess its effectiveness on process measures and clinical outcomes. Both groups received government-supported CKD management training; the intervention group additionally received the CDSS.

## Methods

### Study Design

This cluster randomized clinical trial was conducted at 30 primary care centers in Xicheng District of Beijing, China, to minimize contamination and to align with CDSS implementation. The full protocol is available in Supplement 1. This study followed the Consolidated Standards of Reporting Trials (CONSORT) reporting guideline and was approved by the Ethics Committee of Peking University First Hospital and the Xicheng District Health Commission, which waived informed consent because the intervention posed minimal risk. The waiver also applied to data collection because all data were deidentified and analyzed within a secure local area network.

The study follows a 3-year, 2-phase design (eFigure 1 in Supplement 2). Phase 1 was a 6-month evaluation (June 10, 2023, to December 10, 2023), assessing process measures and short-term clinical outcomes, nested within phase 2 (June 10, 2023, to June 10, 2026), evaluating long-term outcomes. This article reports the preliminary findings of phase 1.

### Participants

Given the variable visit frequency and absence of fixed patient-PCP assignments, the study sample was drawn from all adults visiting the participating centers during a 1-year screening period (March 10, 2022, to March 10, 2023). All participants were enrolled before cluster randomization; subsequent randomization of centers assigned them to the intervention or control group. We included adults (aged ≥18 years) with CKD and 2 or more visits during the screening period. CKD was defined by at least one of the following criteria: (1) 2 estimated glomerular filtration rate (eGFR) values less than 60 mL/min/1.73 m^2^ (CKD Epidemiology equation) obtained at least 90 days apart; (2) confirmed proteinuria, defined as a urine albumin to creatinine ratio of 30 mg/g or greater, urine dipstick 1+ protein or higher, or 24-hour urine protein level of 150 mg or greater (with 2 positive results required ≥90 days apart); or (3) documented CKD codes, supplemented by at least 1 qualifying eGFR or proteinuria meeting the thresholds above. All participants were required to have Beijing health insurance (>91% coverage among primary care patients) to ensure follow-up visits and enable outcome assessment through the Beijing Inpatient Health Insurance Database. Exclusion criteria included pregnancy, malignant tumor, and end-stage kidney disease (maintenance kidney replacement or eGFR <15 mL/min/1.73 m^2^). Secular trends used annual cross-sectional data from all adult patients with CKD treated at participating centers between January 1, 2018, and December 31, 2022, regardless of visit frequency.

### Randomization and Blinding

Stratified cluster randomization by center size (large, medium, or small based on annual visit volume) was performed. Within each category, each center as a cluster was randomized 1:1 to intervention or control. Due to the pragmatic nature of the intervention, blinding was applied only to independent outcome evaluators and statisticians.

### Training

With government support, we conducted a structured training series to enhance CKD management. The initiative began with an on-site mobilization symposium on policy framework, local epidemiology, and evidence-based strategies. Attendees included district health administrators, center directors, and physician representatives. After randomization, certified nephrologists conducted standardized on-site training at each center within 1 month, covering stage-appropriate monitoring, pharmacotherapy, referral criteria, and comorbidity management, as well as the rationale and functionality of the CDSS, with question-and-answer sessions. Technical staff provided parallel training on CDSS operation. The control group received the same government-supported CKD training as the intervention group (mobilization conferences and on-site sessions covering screening, diagnosis, and management) without CDSS instruction.

### CDSS

The CDSS was developed using a PDSA (plan-do-study-act) framework. In the plan phase, semistructured interviews with PCPs identified user needs. The do phase involved a multidisciplinary team (nephrologists, technical experts, and PCP representatives) designing an initial prototype. A 12-month pilot study (September 10, 2021, to September 10, 2022) at an independent primary care center constituted the initial study phase, during which the prototype was tested and refined. Findings informed the act phase, leading to system modifications and the current trial as a subsequent PDSA cycle. Findings from phase 1 will inform future adaptation or redesign of the intervention. Embedded in the electronic health record (EHR) system, the CDSS provided real-time assistance during CKD encounters. Active pop-up alerts were triggered for CKD diagnosis, inappropriate medications, and severe complications, whereas passive recommendations (requiring a click to access) were provided for renoprotective medication initiation (eg, RAASis and sodium-glucose cotransporter 2 inhibitors [SGLT2is]), monitoring schedules, and knowledge base content.

### Data Collection and Follow-Up

Patients were electronically identified via a standardized EHR platform that automatically extracted and harmonized clinical data from all primary care centers in the Xicheng District. Extracted data, including visit dates, demographic characteristics, diagnostic codes, prescribed medications, laboratory results, and blood pressure (BP) measurements, were manually verified and cross-validated under researcher supervision (X.Z., M.H., H.Y.). Data deidentification was performed at the source centers before transmission.

Consistent with the pragmatic design, follow-up was restricted to routine clinical encounters. Records were linked with the Beijing Health Insurance Database for hospitalizations, including diagnoses, mortality status, and costs. CDSS interaction data were recorded through system logs.

### Outcomes

The primary outcome, to be assessed in phase 2, is a 36-month composite of kidney-related (acute kidney injury, progression of kidney disease, or need for dialysis) and cardiovascular (myocardial infarction, stroke, heart failure, or cardiovascular procedures) hospitalizations. This 6-month analysis evaluated changes in process measures (CKD diagnosis, RAASi use, and SGLT-2i use) and clinical outcomes (BP, glycated hemoglobin [HbA_1c_], and low-density lipoprotein cholesterol [LDL-C] control) among participants with at least 1 follow-up visit. Baseline CKD diagnosis was determined by *International Statistical Classification of Diseases and Related Health Problems, Tenth Revision *(*ICD-10*)–coded diagnoses during the screening period; follow-up diagnosis at 6 months was assessed similarly. RAASi and SGLT-2i use was ascertained from prescription records. Optimal control was defined as systolic BP less than 130 mm Hg, diastolic BP less than 80 mm Hg, HbA_1c_ less than 7%, and LDL-C less than 100.4 mg/dL (to convert to millimoles per liter, multiply by 0.0259). Baseline values represented the most recent measurements within the screening period; follow-up values at 6 months were the measurement closest to that time point.

### Qualitative Data Collection and Analysis

PCPs and administrators from intervention centers were recruited through convenience sampling. Two nephrologists (M.H. and H.Y.) conducted semistructured interviews to explore user experience (interface usability, functionality, and clinical utility), implementation determinants (facilitators and barriers), and actionable strategies for CDSS refinements and CKD care enhancements. Interviews were conducted online or in person based on participant preference, using individual or focus group formats. All interviews were audio-recorded with consent, transcribed verbatim using NVivo, version 14 (QSR International), and analyzed through thematic analysis. Key themes were identified through primary analysis by a single coder (M.H.).

### Power

Power analysis was based on the primary outcome of the overall trial (phase 2). On the basis of the Chronic Renal Insufficiency Cohort, which reported a hospitalization rate of 35% in patients with CKD,^12^ we estimated a 20% relative reduction in the intervention group. Assuming an intracluster correlation coefficient of 0.02, a 2-sided α = .05, and 30 centers (15 per group), a sample size of 3240 participants was calculated using the method of Hemming et al^13^ for cluster randomized trials, providing greater than 90% statistical power.

### Statistical Analysis

Within-group changes from baseline to 6 months were presented as rate difference with 95% CIs. The intervention effect was quantified as the between-group difference in these changes, presented as absolute (percentage points with 95% CIs) and relative (odds ratios [ORs] with 95% CIs) values. Outcomes were analyzed using generalized linear mixed models with binomial distribution and logit link. Fixed effects included intervention, time, and their interactions; adjusted models added age, sex, diabetes, hypertension, and cardiovascular disease. A random intercept for cluster accounted for between-center differences with unstructured covariance. All analyses followed the intention-to-treat principle. An as-treated analysis was prespecified if CDSS functionality decreased below 60% but was not triggered because all centers maintained stable operation. Missing data were not imputed; complete-case analyses were conducted using available data at each time point. CDSS engagement was analyzed using descriptive statistics and χ^2^ tests. Analyses were conducted using RStudio, version 2022.02.03 (Posit PBC) with the lme4 package, version 1.1-37. A 2-sided *P* < .05 was considered statistically significant.

## Results

**Figure 1.**
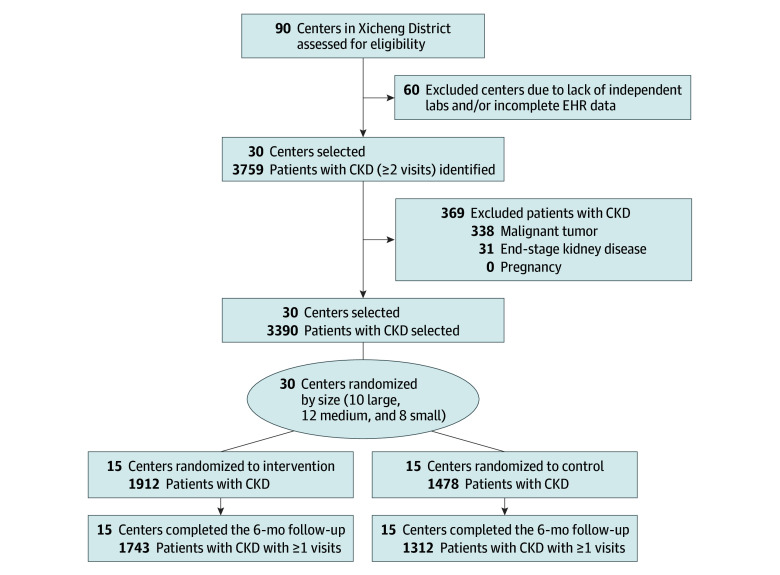
Participant Flow Diagram CKD indicates chronic kidney disease; EHR, electric health record.

Figure 1 shows the flow of participants through the study. A total of 30 primary care centers (10 large, 12 medium, and 8 small) were randomized 1:1 to intervention (n = 15) or control (n = 15). During the selection period, 3390 patients with CKD were enrolled (intervention: 1912; control: 1478). The mean (SD) age was 72.0 (10.2) years, 1509 patients were male (44.5%), and 1881 (55.5%) were female. Mean (SD) eGFR was 67.4 (22.5) mL/min/1.73 m^2^. Baseline data were complete for 3162 patients (93.3%) for BP, 2783 (82.1%) for LDL-C, and 1289 (38.0%) for HbA_1c_, comparable between groups. Table 1 gives the baseline patient-level characteristics; groups were generally comparable, although the intervention group had higher CKD diagnosis rates (1143 of 1912 [59.8%] vs 719 of 1478 [48.6%]). Center-level characteristics are presented in eTable 1 in Supplement 2. This imbalance was primarily driven by the large-center stratum, where diagnosis rates were 48.6% (719 of 1478) in the intervention group vs 62.1% (860 of 1385) in the control group. Observed intracluster correlation coefficients were 0.096 for CKD diagnosis, 0.003 for RAASi use, 0.002 for SGLT-2i use, 0.079 for BP control, and less than 0.001 for HbA_1c_ and LDL-C control.

**Table 1.** Baseline Characteristics of Participants

Characteristic	No. (%) of participants^a^		
	Total (N = 3390)	Intervention (n = 1912)	Control (n = 1478)
Age, mean (SD), y	72.0 (10.2)	71.6 (10.4)	72.5 (9.9)
Sex			
Male	1509 (44.5)	859 (44.9)	650 (44.0)
Female	1881 (55.5)	1053 (55.1)	828 (56.0)
Baseline eGFR, mean (SD), mL/min/1.73 m^2^	67.4 (22.5)	68.8 (22.4)	65.5 (22.4)
CKD stage			
1 (eGFR ≥90 mL/min/1.73 m^2^)	638 (18.8)	362 (18.9)	276 (18.7)
2 (eGFR 60-89 mL/min/1.73 m^2^)	1231 (36.3)	726 (38.0)	505 (34.2)
3 (eGFR 30-59 mL/min/1.73 m^2^)	1383 (40.8)	746 (39.0)	637 (43.1)
4 (eGFR 15-29 mL/min/1.73 m^2^)	138 (4.1)	78 (4.1)	60 (4.1)
Proteinuria	1900/1995 (95.2)	1100/1150 (95.7)	800/845 (94.7)
Missing	1395	762	633
Systolic BP, mean (SD), mm Hg	127 (9)	127 (9)	128 (8)
Missing	228	113	115
Diastolic BP, mean (SD), mm Hg	74 (7)	74 (7)	74 (6)
Missing	228	113	115
HbA_1c_, mean (SD), % of total hemoglobin	7.0 (1.4)	7.0 (1.4)	7.0 (1.3)
Missing	2101	1154	947
LDL-C, mean (SD), mg/dL	100.4 (34.7)	100.4 (38.6)	100.4 (34.7)
Missing, No.	607	314	293
CKD diagnosis	1862 (54.9)	1143 (59.8)	719 (48.6)
Diabetes diagnosis	1909 (56.3)	1088 (56.9)	821 (55.5)
RAASi use	1533 (45.2)	880 (46.0)	653 (44.2)
SGLT-2i use	494 (14.6)	290 (15.2)	204 (13.8)
Statin use	1985 (58.6)	1124 (58.8)	861 (58.3)
BP control (<130/80 mm Hg)	1522/3162 (48.1)	897/1799 (49.9)	625/1363 (45.9)
Missing	228	113	115
HbA_1c_ control (<7%)	772/1289 (59.9)	450/758 (59.4)	322/531 (60.6)
Missing	2101	1154	947
LDL-C control (<100.4 mg/dL)	1550/2783 (55.7)	890/1598 (55.7)	660/1185 (55.7)
Missing	607	314	293

Abbreviations: BP, blood pressure; eGFR, estimated glomerular filtration rate; HbA_1c_, glycated hemoglobin; LDL-C, low-density lipoprotein cholesterol; RAASi, renin-angiotensin-aldosterone system inhibitor; SGLT-2i, sodium-dependent glucose transporters 2 inhibitor.

SI conversion factors: To convert HbA_1c _to proportion of total hemoglobin, multiply by 0.01; LDL-C to mmol/L, multiply by 0.0259

^a^ Unless otherwise indicated.

**Figure 2.**
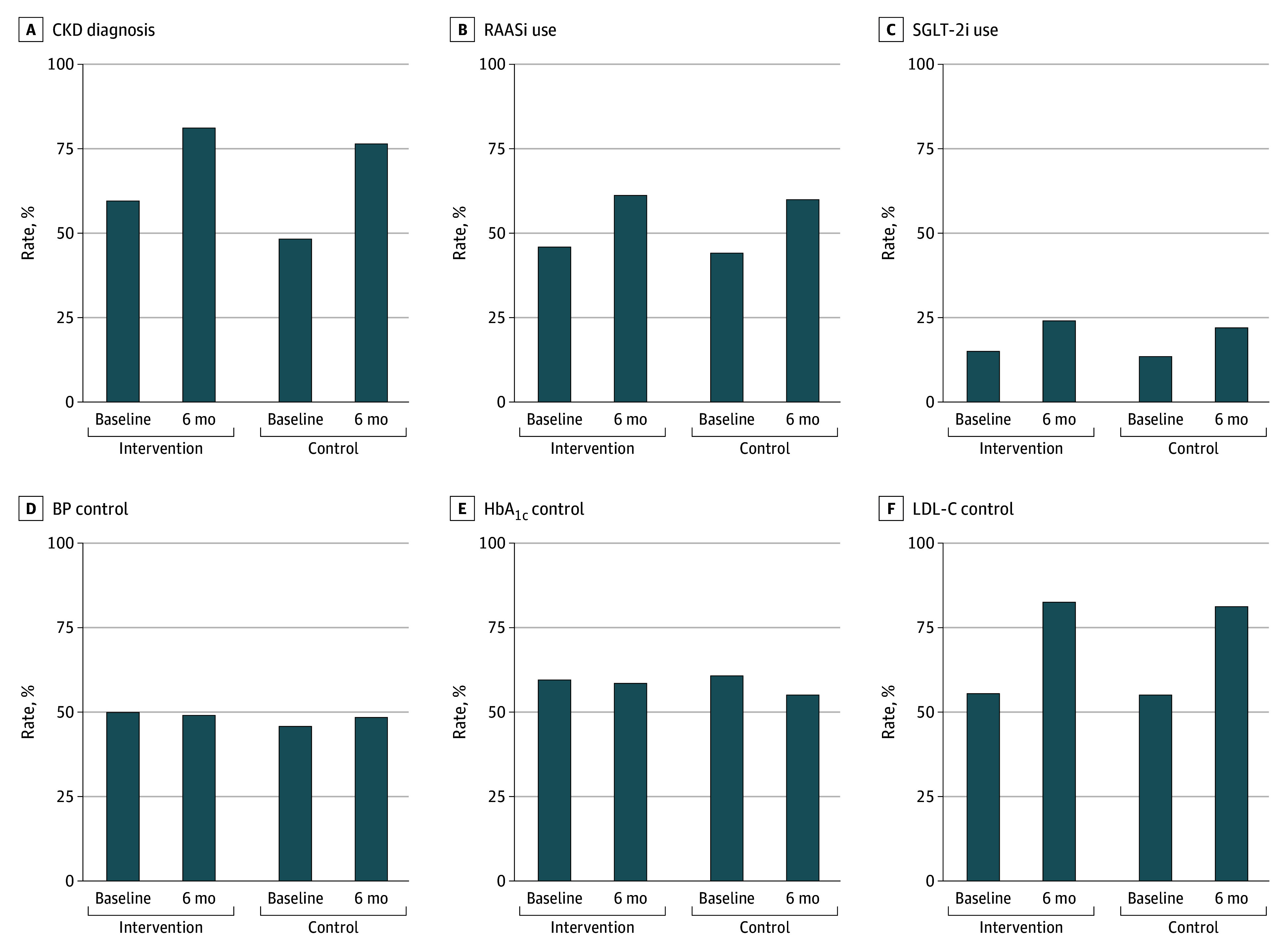
Bar Graph of Process Measures and Clinical Outcomes at Baseline and 6-Month Follow-Up by Study Group BP indicates blood pressure; CKD, chronic kidney disease; HbA_1c_, glycated hemoglobin; LDL-C, low-density lipoprotein cholesterol; RAASi, renin-angiotensin-aldosterone system inhibitor; SGLT-2i, sodium-glucose cotransporter 2 inhibitor.

Follow-up at 6 months was completed by 3055 patients (90.1%), with similar rates between groups (intervention: 1743 [91.2%]; control: 1312 [88.8%]). Among those completing follow-up, data completeness varied by outcome: BP (2941 [96.2%]), LDL-C (1912 [62.6%]), and HbA_1c_ (356 [11.7%]). Figure 2 and Table 2 present within-group changes from baseline to 6 months and the corresponding between-group differences for all outcomes. CKD diagnosis rates increased by 21.4 (95% CI, 18.6-24.3) percentage points in the intervention group and 27.9 (95% CI, 24.4-31.3) percentage points in the control group, with a between-group absolute difference of −6.4 (95% CI, −10.7 to −2.0) percentage points and a nonsignificant relative effect (unadjusted odds ratio [OR], 0.91; 95% CI, 0.72-1.14; adjusted OR [AOR], 0.91; 95% CI, 0.72-1.14). Both groups showed comparable improvements in RAASi use (intervention: 880 of 1912 [46.0%] to 1068 of 1743 [61.3%]; control: 653 of 1478 [44.2%] to 783 of 1312 [59.7%]), SGLT-2i use (290 of 1912 [15.2%] to 423 of 1743 [24.3%] vs 204 of 1478 [13.8%] to 290 of 1312 [22.1%]), and LDL-C control (890 of 1598 [55.7%] to 906 of 1097 [82.6%] vs 660 of 1185 [55.7%] to 665 of 815 [81.6%]), with no significant intervention effects. Neither group achieved significant improvements in BP or HbA_1c_ control. To assess whether these changes reflected intervention effects rather than secular trends, we examined annual historical data from 2018 to 2022 (eFigure 2 and eTable 2 in Supplement 2). During this period, CKD diagnosis rates fluctuated, RAASi use remained stable, and SGLT-2i use increased steadily, whereas clinical control rates showed no sustained improvement. The 6-month improvements observed during the trial exceeded these historical trends.

**Table 2.** Process Measures and Clinical Outcomes at Baseline and 6-Month Follow-Up by Study Group

Measure^a^	Intervention			Usual care			Between-group differences		
	Baseline, No./total No. of patients	6 mo, No./total No. of patients^b^	Within-group changes, percentage points (95% CI)^c^	Baseline, No./total No. of patients	6 mo, No./total No. of patients^b^	Within-group changes, percentage points (95% CI)^c^	Absolute difference, percentage points (95% CI)^d^	Relative difference, OR (95% CI)^e^	
								Unadjusted	Adjusted
CKD diagnosis	1143/1912 (59.8)	1416/1743 (81.2)	21.5 (18.6 to 24.3)	719/1478 (48.6)	1004/1312 (76.5)	27.9 (24.4 to 31.3)	−6.42 (−10.88 to −1.95)	0.91 (0.72 to 1.14)	0.91 (0.72 to 1.14)
RAASi use	880/1912 (46.0)	1068/1743 (61.3)	15.2 (12.1 to 18.4)	653/1478 (44.2)	783/1312 (59.7)	15.5 (11.8 to 19.2)	−0.25 (−5.12 to 4.62)	0.96 (0.79 to 1.19)	0.96 (0.78 to 1.19)
SGLT-2i use	290/1912 (15.2)	423/1743 (24.3)	9.1 (6.5 to 11.7)	204/1478 (13.8)	290/1312 (22.1)	8.3 (5.4 to 11.2)	0.80 (−3.04 to 4.64)	1.02 (0.78 to 1.32)	1.02 (0.78 to 1.32)
BP control	897/1799 (49.9)	824/ 1682 (49.0)	−0.9 (−4.2 to 2.5)	625/1363 (45.9)	612/1259 (48.6)	2.8 (−1.1 to 6.6)	−3.63 (−8.69 to 1.44)	0.88 (0.71 to 1.09)	0.88 (0.71 to 1.09)
HbA_1c_ control	450/758 (59.4)	123/ 211 (58.3)	−1.1 (−8.6 to 6.4)	322/531 (60.6)	80/145 (55.2)	−5.5 (−14.6 to 3.6)	4.39 (−7.41 to 16.20)	1.25 (0.77 to 2.04)	1.22 (0.74 to 2.01)
LDL-C control	890/1598 (55.7)	906/1097 (82.6)	26.9 (23.6 to 30.2)	660/1185 (55.7)	665/815 (81.6)	25.9 (22.0 to 29.8)	1.00 (−4.11 to 6.10)	1.10 (0.83 to 1.46)	1.10 (0.83 to 1.46)

Abbreviations: BP, blood pressure; eGFR, estimated glomerular filtration rate; HbA_1c_, glycated hemoglobin; LDL-C, low-density lipoprotein cholesterol; OR, odds ratio; RAASi, renin-angiotensin-aldosterone system inhibitor; SGLT-2i, sodium-dependent glucose transporters 2 inhibitor.

^b^ For 6-month outcomes, denominators reflect patients with follow-up visits (1743 in the intervention group and 1312 in the control group). Denominators for BP, HbA_1c_, and LDL-C vary due to testing patterns.

^c^ Within-group changes are calculated as the difference between 6-month and baseline values, expressed in percentage points.

^d^ Absolute difference is the between-group difference of the within-group changes, expressed in percentage points.

^e^ Relative difference is presented as ORs comparing the intervention effect to control, estimated from generalized linear mixed models. Adjusted ORs were adjusted for age, sex, and comorbidities (eg, diabetes, hypertension, cardiovascular disease).

^e^ Missing rates at 6 months are as follows: BP control, 114 (3.8%; 61 [3.5%] in the intervention group and 53 [4.0%] in the usual care group); HbA_1c_ control, 2699 (88.3%; 1532 [87.9%] in the intervention group and 1167 [88.9%] in the usual care group); and LDL-C control, 1143 (37.4%; 646 [37.1%] in the intervention group and 497 [37.9%] in the usual care group).

During the 6-month intervention, 735 clicks for passive recommendations were recorded. Among patients with an eGFR less than 45 mL/min/1.73 m^2^, metformin use was lower in the intervention group (41 of 251 [16.3%] vs 55 of 227 [24.2%]; *P* = .03). Among those with an eGFR less than 60 mL/min/1.73 m^2^, nonsteroidal anti-inflammatory drug use was also lower in the intervention group (39 of 751 [5.2%] vs 52 of 626 [8.3%]; *P* = .02). No adverse events related to the intervention were reported, and potential medication-related harms (eg, hyperkalemia, acute kidney injury, hypotension, and urinary tract infections) were similar between groups.

Interviews with 32 PCPs and 8 administrators across 15 intervention centers highlighted several key findings. Participants trusted the CDSS, crediting its design by a joint team of nephrologists and PCPs. Drug safety alerts were considered the most clinically useful feature because they addressed immediate risks. PCPs reported hesitancy to engage in proactive therapeutic adjustment during time-limited visits because renoprotective medications offer no short-term observable benefits. Even when indicated, initiating use of RAASis raised concern about worsening kidney function or hypotension—potential short-term harms that create patient ambivalence. Key facilitators included on-demand access to nephrology expertise, linkage of CDSS use to performance evaluation, and high-priority alerts. Main barriers included the absence of timely feedback, inability to observe patient improvement, difficulty communicating new CKD diagnoses, and hesitancy to modify treatment when comanaging with nephrologists.

## Discussion

This cluster randomized clinical trial represents the first clinical evaluation of a CDSS for CKD management in Chinese primary care. Across 6 months, both groups showed comparable improvements in CKD diagnosis, RAASi or SGLT-2i use, and LDL-C control that exceeded historical trends. However, the CDSS itself did not demonstrate an additive benefit beyond these parallel gains. On the basis of these findings, the original phase 2 plan has been reassessed and will not proceed as initially designed. Instead, these results are being used within the PDSA framework to inform future interventions.

Despite the World Health Organization’s classification of CKD as a priority noncommunicable disease,^14^ evidence of effective primary care interventions remains limited. Previous CDSS studies,^9,15,16,17,18,19^ predominantly conducted in high-income countries, have demonstrated only modest clinical benefits. For instance, audit-based interventions achieved minimal BP reduction (2.41 mm Hg),^15^ whereas a systematic review found 5.8% improvement in targeted care processes.^16^ These limited effects may reflect high baseline care standards; data from the UK suggest only 3.9% of late dialysis presentations were avoidable,^17^ which may explain why eGFR surveillance failed to reduce late referrals in a recent UK trial.^9^ In contrast, China’s health care environment is characterized by high CKD prevalence with low awareness, nephrologist shortages, and undertrained PCPs,^18,19^ creating substantial opportunities for intervention impact and motivating our pragmatic design.

Our study provided nephrologist-delivered training to both groups, distinguishing it from prior studies in which training was embedded within the intervention.^8,20^ This likely explains the comparable improvements between groups and the absence of an independent CDSS effect. In China, CKD underdiagnosis is fundamentally linked to suboptimal PCP training,^21^ with underawareness rather than detection capacity identified as the primary barrier.^22^ In this context, dedicated training alone may yield substantial improvement, particularly when addressing fundamental knowledge gaps. Beyond knowledge transfer, these gains may also reflect enhanced care coordination. Consistent with prior work,^23,24^ accessible nephrologist support remains essential for PCPs managing CKD. Onsite training facilitated nephrologist-PCP communication, addressing shared care roles and individualized medication adjustment,^25^ and may have contributed to the parallel improvements.

Despite gains in CKD diagnosis, HbA_1c_ control remained suboptimal, with low monitoring rates, and improvements in RAASi and SGLT-2i use were modest. This diagnosis-action gap may be attributed to therapeutic inertia. Under heavy workloads, PCPs deprioritized initiating renoprotective medications, viewing them as nonurgent and potentially creating short-term harms (eg, hypotension and acute kidney injury) that may outweigh unobservable long-term benefits. In contrast, the CDSS effectively reduced inappropriate prescribing, suggesting safety-focused alerts that prevent immediate harm were more readily adopted. However, our CDSS provided only passive recommendations for medication initiation, which physicians rarely viewed, and such low engagement was insufficient to overcome therapeutic inertia. These findings highlight the need for risk-stratified, tiered alerts that escalate as needed, with dynamic adjustment based on regular feedback.

These contrast patterns (success in reducing inappropriate prescribing with active safety alerts but limited impact of passive reminders on proactive care) align with broader challenges. User engagement is crucial for successful adoption: one noninterruptive design demonstrated minimal engagement (122 clicks by 57 PCPs during 6 months) with no improvement in CKD monitoring,^26^ whereas another achieved a 74% opening rate that correlated with improved awareness.^10^ Our study builds on these insights by integrating PCP input during development and securing administrative support,^8^ yet engagement barriers persist. Time pressure in chronic disease management^27^ leads PCPs to prioritize high-risk scenarios (eg, medication safety alerts) over recommendations with delayed returns. This is supported by a prior finding that only 50% of PCPs report comfort in establishing a CKD diagnosis.^28^ Enhancing engagement requires risk-stratified alerts, targeted education, and workflow integration with dynamic feedback.

### Strengths and Limitations

As the first trial, to our knowledge, to evaluate CDSS-assisted CKD management in Chinese primary care, our study has notable strengths. We established technical feasibility of an EHR-embedded CDSS for patient identification and intervention delivery. Through proactive PCP engagement, we identified key education needs and systemic challenges. The inclusion of historical control data confirmed that improvements exceeded secular trends. Our policy-aligned approach facilitated implementation and supported scalability. The cluster randomized design accounted for the absence of fixed patient-PCP relationships while leveraging existing EHR data to align with clinical workflows, making the intervention well suited for primary care.

This study also has limitations. First, absence of active follow-ups resulted in incomplete outcome data, although linkage with inpatient health insurance databases captured significant events. Second, this 6-month analysis was not separately powered and was intended as an exploratory interim assessment to guide PDSA-based refinements; findings should be interpreted with caution. Third, 60 of 90 centers were excluded due to infrastructural limitations, which may limit generalizability. Fourth, substantial cluster size variation was not accounted for in sample size calculation. Fifth, identification of medication contraindications was limited by inconsistent documentation. Sixth, relevant medications (eg, statins) were not included as outcomes. Seventh, baseline imbalances driven by uneven distribution of large centers despite stratified randomization may attenuate the estimated intervention effect.

## Conclusions

This cluster randomized trial found no additive benefit of a CKD CDSS beyond concurrent training and policy support. However, the intervention successfully reduced inappropriate prescribing through safety alerts, demonstrating the potential of interruptive alerts. These findings suggest that in primary care settings with suboptimal baseline conditions, targeted education and policy support remain foundational, whereas CDSS should integrate dynamic feedback and interruptive alerts to optimize effectiveness.

## Notes

Supplement 1.
Trial Protocol

Supplement 2. **eFigure 1**. Study Design **eFigure 2.** Historical Trends in Process Measures and Clinical Outcomes at Participating Study Sites (2018–2022) Prior to CDSS Intervention. **eTable 1.** Characteristics of Participating Centers and Primary Care Physicians **eTable 2.** Historical Trends in Process Measures and Clinical Outcomes at Participating Study Sites (2018–2022) Prior to CDSS Intervention.

Supplement 3.
Data Sharing Statement
