# Supplementary material for: Clinical Decision Support for Chronic Kidney Disease in Primary Care: A Cluster Randomized Clinical Trial
Source: JAMA Netw Open. 2026 May 8;9(5):e2611112. doi: 10.1001/jamanetworkopen.2026.11112 (PMC13156789; doi:10.1001/jamanetworkopen.2026.11112)
Supplement: Supplement 1. — Trial Protocol [file jamanetwopen-e2611112-s001.pdf]

A Clinical Decision Support System for Managing Chronic Kidney Disease in Primary Care: A Multicenter Cluster Randomized Controlled Trial

Trial sponsor: Peking University First Hospital

date: June 20, 2022

Version number: V1.0

1. Background

Chronic kidney disease (CKD) represents a significant and escalating global public health challenge, with a particularly heavy burden in China that affects approximately 120 million individuals, the majority of whom are in the early stages (1-3) where intervention is most effective. However, despite this high prevalence, CKD remains substantially underdiagnosed and inadequately managed in primary care settings where most patients initially present. This considerable care gap persists even though primary care physicians (PCPs) serve as the frontline managers, a situation compounded by critically low public disease awareness (12.5%)—largely due to the disease's insidious onset—and insufficient knowledge among PCPs themselves, as evidenced by only 24.9% awareness rate of fundamental CKD concepts, staging, and management principles among those in Beijing. This dual deficit in awareness creates a significant imbalance between the existing healthcare supply capabilities and the population's needs for effective CKD prevention and management.

Digital tools for CKD management in primary care have evolved from basic eGFR reporting to comprehensive clinical decision support systems (CDSS). While the introduction of eGFR reporting improved renal referral rates, it showed limited impact on key process measures like renin-angiotensin-aldosterone system inhibitors (RAASi) use. Subsequent generations of CDSS, incorporating educational modules and multifactorial interventions, have yielded only modest or inconsistent improvements in care processes and patient outcomes in high-income countries. This limited effectiveness may be attributable to the already high baseline standard of CKD management in these settings, thereby constraining the potential for substantial further gains. In contrast, the considerable CKD burden, suboptimal management in primary care, and constrained specialist nephrology resources in China provide an ideal environment for CDSS implementation to enhance care quality. Despite this potential, no prior trials have evaluated the effectiveness of such interventions in Chinese primary care system.

This study is designed to address these gaps by developing a CKD-specific CDSS, contextualized for China's primary care setting through a multidisciplinary team that includes PCPs, nephrologists, and other specialists. A subsequent multicenter cluster-randomized trial will be conducted to evaluate the effectiveness of this CDSS versus usual care on both process measures and clinical outcomes. The CDSS will provide comprehensive support, encompassing screening, diagnosis, and management guidance. To isolate the effect of CDSS from that of general CKD training, government-supported CKD training will be provided to both study arms. This innovative approach aims to validate a scalable model for early CKD prevention and

control in Chinese communities, thereby ultimately generating evidence-based support to alleviate the CKD disease burden.

2. Research Hypothesis and Research Objectives

2.1 Research Hypothesis

Our central hypothesis is that the implementation of a tailored CDSS will optimize PCPs' medical behaviors—including screening, diagnosis, and treatment—which will, in turn, lead to improved patients’ outcomes and a consequent reduction in the burden of CKD in the community setting.

2.2 Research Objectives

2.2.1 Primary Objectives

1. To assess the effect of the CDSS on the composite endpoint of kidney-related and cardiovascular hospitalization rates among community CKD patients.

2. To evaluate the impact of CDSS on kidney-related hospitalization rate among community patients at high risk for CKD.

2.2.2 Secondary Objectives

Process Measures:

1. To assess the effect of the CDSS on the underdiagnosis of CKD, as measured by the rate of appropriate ICD-coded documentation in patient records.

2. To examine the change in the utilization rates of renoprotective medications (RAASi and sodium-dependent glucose transporters 2 inhibitor [SGLT2i]) among CKD patients.

3. To evaluate the impact of the CDSS on the screening rate for CKD (SCr and/or urine protein testing) among patients at high risk for CKD.

4. To determine the effect of the CDSS on the referral rate of CKD patients who meet guideline-based criteria for nephrology consultation.

Clinical Outcome Measures:

5. To evaluate the effect of the CDSS on blood pressure control (target: <130/80 mmHg) among patients with CKD.

6. To assess the impact of the CDSS on glycemic control (target: HbA1c <7%) among patients with diabetic kidney disease.

7. To examine the effect of the CDSS on lipid management (target: LDL-C <2.6 mmol/L) among patients with CKD.

Health Economic and Implementation Outcomes:

8. To analyze the cost-effectiveness of the CKD screening and management program within the community setting.

9. To conduct a comprehensive process evaluation of CDSS implementation using the RE-AIM framework.

3. Research Design

This study employs a multicenter, parallel-group, cluster-randomized controlled trial design. A total of 30 community health centers in the Xicheng District of Beijing will be recruited, with each center constituting a cluster. The study population consist of adult CKD patients and patients at high-risk for CKD-who are regularly managed at these centers, defined as having two or more visits documented in the Electronic Health Record (EHR) system during a designated 12-month screening period. Baseline characteristics, including CKD diagnosis (based on ICD codes) and clinical measures (e.g., the most recent blood pressure, HbA1c, and LDL-c values), were all ascertained from data recorded during this screening period. These 30 clusters will be stratified by their annual patient visit volume (large, medium, small) and subsequently randomized in a 1:1 ratio to either the intervention or control group. Following randomization and prior to the formal initiation of the intervention, both groups will receive the government-supported structured training. The intervention group will then implement a CKD-specific CDSS integrated into their existing EHR platforms, in addition to providing government-supported structured training. The control group will continue with usual care, also supplemented by the same government-supported training to isolate the effect of the CDSS itself. The trial will employ a two-phase evaluation scheme: a 6-month initial phase assessing the impact of the CDSS on process measures (CKD diagnosis, RAASi/SGLT-2i use) and short-term clinical control rates (blood pressure, HbA1c, LDL-c), and the final 36-month phase evaluating its effects on the primary composite endpoint of kidney and cardiovascular hospitalization rates, alongside a comprehensive health economic analysis, which will be conducted through linkage with the centralized Beijing Health Insurance Database.

3.1 Research Subjects

3.1.1 Study Population

The study population comprise adult patients managed across 30 community health centers in Xicheng District, Beijing (**Table 1**). To ensure sufficient data for follow-up, patients were selected from all visits to these centers during a one-year screening period (March, 10, 2022 – March, 10, 2023), with the inclusion criterion of having at least two visits during this period. The study is primarily focused on CKD patients, while analysis will include a cohort of high-risk population for developing CKD. ICD codes for defining diseases in study population inclusion/exclusion criteria are summarized in **Table 2**.

3.1.2 CKD Patients

3.1.2.1 Inclusion Criteria

1. Age≥18 years

2. Diagnosis of CKD, defined as meeting at least one of the following criteria:

(1) Two estimated glomerular filtration rate (eGFR) values <60 mL/min/1.73m² (calculated using the CKD-EPI equation), obtained ≥90 days apart.

(2) Confirmed proteinuria, defined as two positive results ≥90 days apart for either urine albumin-to-creatinine ratio (UACR) ≥30 mg/g, urine dipstick ≥1+, or 24-hour urine total protein (UTP) ≥150 mg.

(3) Documented CKD diagnostic codes in the EHR, supplemented by at least one qualifying eGFR or proteinuria result meeting the above thresholds.

3. Beijing medical insurance residents.

3.1.2.2 Exclusion Criteria

1. Pregnancy: Patients with pregnancy/perinatal ICD diagnosis codes in EHR.

2. Malignancy

3. End-stage kidney disease: maintenance kidney replacement or kidney transplantation by ICD codes, or eGFR<15 ml/min/1.73m²

3.1.3 High-risk Patients for CKD

3.1.3.1 Inclusion Criteria

1. Age≥18 years

2. No established diagnosis of CKD

3. Presence of at least one of the following risk factors:

(1) Type 2 diabetes mellitus (T2DM) (ICD-coded)

(2) Hypertension (ICD-coded)

(3) Cardiovascular disease (ICD-coded)

(4) Obesity: body mass index (BMI) ≥28.0 kg/m² per Chinese expert consensus, or relevant ICD-10 codes.

(5) History of AKI/AKD: meeting either KDIGO SCr criteria or a clinical diagnosis, as detailed in **Table 3**

(6) Kidney stones (ICD-coded)

(7) Family history of kidney disease

(8) Advanced age (>60 years)

3.1.3.2 Exclusion Criteria

Identical to the exclusion criteria specified in section 3.1.2.2 for the CKD population.

3.2 Study Interventions

This study employs a multi-faceted intervention strategy, comprising a structured training program for PCPs and the implementation of a CDSS within the EHR infrastructure of community health services in Beijing Xicheng District, with administrative support from the Beijing Municipal Government and Xicheng District Health Commission.

3.2.1 CDSS

The CDSS will be developed using a Plan-Do-Check-Act framework and will be integrated into the existing EHR system. In the Plan phase, semi-structured interviews with PCPs will be conducted to identify user needs. The Do phase will involve a multidisciplinary team (including nephrologists, technical experts, and PCP representatives) in designing an initial prototype. A pilot study at an independent primary care center will then be conducted as the Check phase, during which the system will be iteratively refined. Finally, the Act phase will consist of evaluating the system's effectiveness and implementation feasibility through the main clinical trial described above. The CDSS is designed to provide non-intrusive, real-time clinical decision support during patient encounters. The key functionalities deployed in the intervention arm include:

1. Automated case identification & risk stratification: The system automatically identifies patients with or at risk of CKD through structured diagnostic codes and laboratory data.

2. Renal referral for patients without prior diagnosis of CKD

(1) Rapid decline in renal function meeting the AKD criteria (**Table 3**).

(2) Presence of refractory hypertension: defined as blood pressure (BP) >140/90 mmHg while prescribed ≥3 antihypertensive medications, or being prescribed ≥4 classes of antihypertensive medications.

(3) Persistently proteinuria, hematuria or eGFR <60 mL/min/1.73m² (two consecutive abnormal results obtained ≥90 days apart)

3. Automated CKD staging (stages 1-5)

4. Structured management protocols for diagnosed CKD patients:

(1) Stage-specific monitoring of renal function (e.g., eGFR, UACR every 3 month).

(2) Complication management alerts for identification, evaluation and management targets based on thresholds defined as follows: hypertension (BP >130/80 with proteinuria or >140/90 without), CKD-mineral and bone disorder (CKD-MBD) (corrected Ca^{2+} <2.2 mmol/L; PO_4^{3-} >1.61 mmol/L), hyperkalemia (K^+ >5.5 mmol/L), metabolic acidosis (pH <7.25 or HCO_3^- <22 mmol/L), anemia (Hb <120 g/L [Male] / <110 g/L [Female]), and dyslipidemia (LDL-C >1.8 mmol/L).

5. Renal referral for CKD patients presenting with following indications:

(1) Advanced CKD (stage 4-5)

(2) With any of the following complications: rapid eGFR decline meeting AKD criteria; persistent hematuria (≥20 RBCs/HPF, ≥80 RBCs/μL, or occult blood ≥2+) on consecutive tests; refractory hypertension; hypoproteinemia (serum albumin <35g/L); CKD-MBD; severe anemia (Hb <100 g/L); significant proteinuria (UACR ≥300 mg/g

or UTP ≥1.0 g/day, or ≥0.5 g/day in diabetics); or persistent hyperkalemia (K+ >5.5 mmol/L after initial treatment).

6. Treatment recommendations for CKD patients:

(1) SGLT2is are recommend as first-line treatment for patients with T2DM and an eGFR ≥30 mL/min/1.73 m².

(2) GLP-1 RAs are introduced for patients with T2DM and suboptimal glycemic control on metformin and SGLT2i, with eGFR ≥15 mL/min/1.73 m².

(3) RAASi are recommended for patients with proteinuria and/or hypertension, contingent upon the following criteria: eGFR ≥30 mL/min/1.73 m² (within the past 6 months), serum K^+ <5.5 mmol/L (within the past 6 months), and absence of bilateral renal artery stenosis.

(4) Dosing for all relevant medications should be adjusted according to renal function, as specified in **Table 4.**

3.2.2 Training Program

With administrative support from the Beijing Municipal Government and Xicheng District Health Commission, we will conduct a structured training series to enhance CKD management capabilities. The initiative will begin with an on-site mobilization symposium, which presents the policy framework for the CKD quality improvement initiative, reviews epidemiological data on the CKD burden in Xicheng District, and emphasizes evidence-based management strategies. Participants of the symposium include key district health administrators, directors of primary care centers, and designated physician representatives across the district.

Following randomization, certified nephrologists will conduct standardized on-site training sessions at each center. These sessions will focus on clinically actionable CKD management strategies and include dedicated Q&A opportunities for PCPs. The curriculums will cover stage-appropriate monitoring intervals, evidence-based pharmacotherapy, nephrology referral criteria, and comorbidity management. At intervention centers, technical staffs will provide concurrent training to PCPs on the operation and workflow integration of the CDSS.

3.3 Study Outcomes

3.3.1 Main Outcomes

1. Hospitalization rates for kidney-related (acute kidney injury, progression of kidney disease, or requiring dialysis) or cardiovascular events including myocardial infarction, stroke, heart failure, or cardiovascular procedures) among CKD patients.

2. Hospitalization rates for kidney-related events in patients at high-risk for CKD

Data sources for main outcomes: Electronic medical record systems of community medical institutions and Beijing medical insurance claims database.

3.3.2 Secondary Outcomes

1. Changes in CKD diagnosis rate: the difference in the proportion of CKD patients with an ICD-code for CKD documentation between the follow-up and baseline periods.

2. Changes in utilization of renoprotective medications: the difference in prescription rates of RAASi and SGLT2i among CKD patients between the follow-up and baseline periods, reported seperately.

3. Changes in screening rate for CKD: the difference in the proportion of patients at high risk for CKD who complete SCr and/or urine protein testing between the follow-up and baseline periods.

4. Changes in renal referral rate: the difference in the proportion of CKD patients meeting criteria for nephrologist consultation referral who are actually referred.

5. Changes in blood pressure control rate: the difference in proportion of CKD patients achieving a target of <130/80 mmHg, based on available tests.

6. Changes in glycemic control rate: the difference in the proportion of CKD patients achieving a HbA1c <7%, based on available tests.

7. Changes in lipid control rate: the difference in the proportion of CKD patients achieving a LDL-C <2.6 mmol/L, based on available tests.

8. Cost-effectiveness of the CKD-CDSS: economic evaluation of the community-based CKD screening and management program.

9. CDSS implementation process: process evaluation using the RE-AIM framework (**Table 5**) to understand the implementation of this complex intervention. This evaluation examines the factors influencing outcomes across five dimensions—Reach, Effectiveness, Adoption, Implementation, and Maintenance—at both the individual and organizational levels. An initial phase, structured questionnaires for community institutions and physicians (**Table 6**) will be used to inform the timely optimization of the CDSS and its implementation strategy.

3.4 Research Methods

3.4.1 Cluster Randomized Controlled Trial Design

This study employs a multicenter, parallel, pragmatic cluster randomized controlled trial (cRCT) design, implemented across 30 primary care centers in Xicheng District, Beijing. A cluster design is chosen to minimize contamination between treatment arms and to align with the natural unit of CDSS implementation at center level. Each of the 30 community health service centers/stations constitutes a cluster.

3.4.2 Randomization and Intervention Allocation

Stratified cluster randomization is employed at center level. Prior to the study, the 30 centers are stratified into three categories based on annual outpatient visit volume: large (≥10,000 visits/year), medium (1,000–9,999 visits/year), and small (<1,000

visits/year). Within these strata, an independent statistician uses a computerized system to randomly assign centers in a 1:1 ratio to the intervention group (CDSS plus usual care) or the control group (usual care alone). Randomization results are concealed until the start of the intervention assignment.

3.4.3 Training Period

Prior to the formal study period, a government-supported training program will be implemented. Following randomization, nephrologists will provide standardized on-site training at all centers on CKD management. At intervention centers, technical staff will additionally train PCPs on CDSS use and integration. This will be followed by a dedicated run-in period during which the CDSS is activated and PCPs receive on-site support; data from this phase will be excluded from the final analysis to establish routine system use.

3.4.4 Study Duration and Phases

The 36-month intervention period are divided into two phases: (1) an initial six-month evaluation (Phase 1) for process measures and short-term clinical outcomes, and (2) a three-year evaluation (Phase 2) for long-term outcomes, with a composite of kidney and cardiovascular hospitalization rates as the primary endpoint.

3.4.5 Blinding

Due to the pragmatic nature of the intervention, blinding of participating PCPs and patients is not feasible. However, outcome assessors and statisticians will be blinded to group allocation throughout the analysis.

3.4.6 Informed Consent

The study received ethical approval from the Peking University First Hospital (2022 Study 489) and the Xicheng District Health Commission (2021 Study 104). The ethics committee granted a waiver for individual patient informed consent, as the intervention was implemented at center level, posed minimal risk by adhering to guideline-based care, and did not alter routine care. This waiver was granted on the condition that we obtained prior administrative approval from all involved community health authorities.

3.4.7 End of the Trial

The trial is planned to conclude 36 months. Upon completion, all centers will be considered to have fulfilled the trial requirements, and the study database will be locked. A trial completion notice will be submitted to the ethics committee. Premature termination may occur under the following circumstances: mandatory request from the ethics committee, recommendation from the data monitoring committee, or discontinuation of trial funding.

3.5 Data Management

Study data are generated from the EHR and LIS of community medical centers in Xicheng District, Beijing. All data are managed and stored by the “Joint Laboratory” established collaboratively by the Xicheng District Smart Health Research Center and Peking University First Hospital for community health and medical big data research.

3.5.1 Data Collection and Management

Prospective data collection will be conducted via the Xicheng District Health Commission data platform linked to the CDSS application server. Following community-developed CDISC-CRF standards, EHR and LIS data will be automatically extracted to generate electronic case report forms (eCRF). After privacy-preserving de-identification, data were synchronized daily (T+1) via a dedicated medical network to the Joint Laboratory to create the research dataset. The PostgreSQL-based research database includes structured domains for patient visits, diagnoses, prescriptions, clinical notes, vital signs, and laboratory reports, with standardized coding applied to diagnoses, medications, and laboratory data to enhance research usability.

The study endpoint—hospitalization rates for kidney-related or cardiovascular events among CKD patients—will be assessed by linking with the Beijing Municipal Inpatient Medical Insurance Database. This database provides comprehensive and auditable hospitalization records for Beijing-insured patients admitted to tertiary hospitals, including demographics, treatments, clinical diagnoses (ICD-10 coded), costs, and department information.

3.5.2 Data Governance

Raw data collected from community CDSS servers will undergo comprehensive governance to ensure suitability for research and eCRF generation. Key steps include dictionary normalization, cleaning of unstructured text, semantic parsing and association, and systematic data quality control.

3.5.3 Data Security and Quality Control

The data management team plans to use several security steps to protect information, including firewalls, systems to prevent data leaks, network monitoring, and activity logs. Data quality will be checked during the entire process—from collection and cleaning to merging and updating. Automated tools will be adopted to complete missing values and correct errors to ensure that the data are complete, standardized, consistent, and accurate. A Data and Safety Monitoring Committee (DSMC) carries out regular quality checks, provides feedback, creates quality reports, and helps to resolve any data issues promptly.

3.5.4 Data Usage

This study will strictly follow national and industry rules for data security. All researchers accessing data are required to sign confidentiality agreements, managed

by the DSMC. Electronic audit trails will record all data entries and modifications, including reasons, timestamps, and user identifiers.

3.6 Statistical Analysis

3.6.1 Sample Size and Statistical Power

The primary outcome is a composite hospitalization rate for kidney-related (acute kidney injury, progression of kidney disease, or requiring dialysis) or cardiovascular events (myocardial infarction, stroke, heart failure, or cardiovascular procedures). Based on data from the US CRIC cohort, the all-cause hospitalization rate for CKD patients is 35.0 per 100 person-years. The sample size calculation aims to detect a 20% reduction in this rate following 36 months of CDSS intervention, compared to usual care. The calculation is performed using cluster randomization tools (online calculator at https://clusterrcts.shinyapps.io/rshinyapp/) with the following parameters: 30 randomized centers (15 per arm), an intraclass correlation coefficient of 0.02, and a significance level of 0.05. A total sample size of 3,240 participants is estimated to provide 90% statistical power (**Figure 1)**.

3.6.2 Data Analysis

3.6.2.1 Analysis Principles

Categorical variables will be described as frequencies and percentages. Continuous variables will be summarized as means with standard deviations or as medians with interquartile ranges, based on their distribution. The primary outcome of hospitalization rate will be analyzed using generalized linear mixed models (GLMMs). Both unadjusted and adjusted estimates will be presented； adjusted analyses will include age, sex, comorbidities (including diabetes, hypertension, and cardiovascular disease), and baseline eGFR. Other secondary outcomes will be analyzed using the same approach but without adjustment for baseline eGFR.

For missing data, if the proportion of missingness is below 20%, continuous variables will be imputed using regression-based methods and categorical variables using predictive mean matching. Any variable with over 20% missing values will be excluded from the analysis.

All analyses will adhere to the intention-to-treat (ITT) principle. Given the pragmatic trial setting, a decline in adherence is anticipated. Should adherence to the CDSS intervention fall below 60%, a secondary as-treated (AT) analysis will be performed as pre-specified. All statistical analyses will be conducted using RStudio (v2022.02.03).

3.6.2.2 Interim Analysis

A pre-specified interim analysis is planned at 6 and 12 months after intervention initiation, evaluating the impact of the CDSS on secondary endpoints, including process-of-care measures and short-term clinical outcomes. The primary endpoint—

the composite rate of kidney- and cardiovascular-related hospitalizations—will be assessed after the full three-year follow-up period.

3.6.2.3 Health economics evaluation

An economic evaluation of the CDSS intervention compared to usual care will be performed. Cost data for clinical events will be sourced via linkage with Beijing medical insurance database. Probabilities for disease progression to renal replacement therapy and cardiovascular events will be derived from the literature and analysis of insurance data. We will develop a Markov model using TreeAge Pro software to estimate incremental life years and quality-adjusted life years (QALYs) gained. Furthermore, sensitivity analyses will be conducted to explore the influence of age, different high-risk patient groups, and CKD stages on the results.

3.6.2.4 Qualitative Data Analysis

Qualitative data will be collected through semi-structured interviews with primary care physicians and administrators from intervention centers. Interviews will explore user experience (e.g., usability, utility), implementation determinants (e.g., facilitators and barriers), and actionable strategies for system improvement. Thematic analysis will be performed on verbatim transcripts using NVivo software to identify key themes through an iterative, systematic coding process.

4. Research Quality Control

As a pragmatic clinical trial, this study will focus its quality control efforts on two key areas: the implementation of the intervention and the management of study data. Following randomization, PCPs at all participating centers will receive standardized training on the trial procedures. Those in the intervention group will receive additional, comprehensive training on the use of the CDSS. In contrast, PCPs in the control group will not be introduced to the CDSS and will only receive training pertinent to routine patient management. This will ensure a consistent understanding of the processes among all sites. Throughout the trial, centers will be required to adhere strictly to the protocol specifications regarding randomization, allocation concealment, and participant eligibility. To maintain the quality of CDSS implementation in real-world settings, a three-tier management system will be established. This system will enable weekly feedback, evaluation, and verification of CDSS-related clinical quality indicators at each community site, allowing for timely identification and continuous improvement of any operational issues.

5. Research Organization and Management

The Principal Investigator (PI) will assume overall responsibility for the conduct of the study. The PI will establish an Academic Committee and a Trial Management Group (TMG) to collaborate with relevant stakeholders, including community health

managers, in overseeing all aspects of the trial. Responsibilities of the PI refer to preparation of the clinical trial application (including the study protocol and ethics submissions), site initiation and training, quality control, data management, statistical analysis, and study reporting. All trial-related documents will be accurately recorded and maintained in the trial master file.

6. Other Provisions

6.1 Study Registration

The trial will be registered with the Chinese Clinical Trial Registry Chi CTR (http://www.chictr.org.cn)

6.2 Publication Policy

The primary trial results will be credited collectively to all co-investigators, study coordinators, and participating institutions. If named authorship is required, the writing committee chair will be listed first, followed by other contributors in alphabetical order. All trial investigators will be listed in the main publication. Findings from this study will be disseminated through presentations at international conferences and publications in peer-reviewed scientific journals.

6.3 Intellectual Property

All results, data, and documents—in any form and whether derived directly or indirectly from this research—will be the property of the PI. The PI shall retain the right to use and develop these research outcomes without restriction, and will hold all relevant intellectual property rights.

Figure 1. Calculation of Cluster Size.

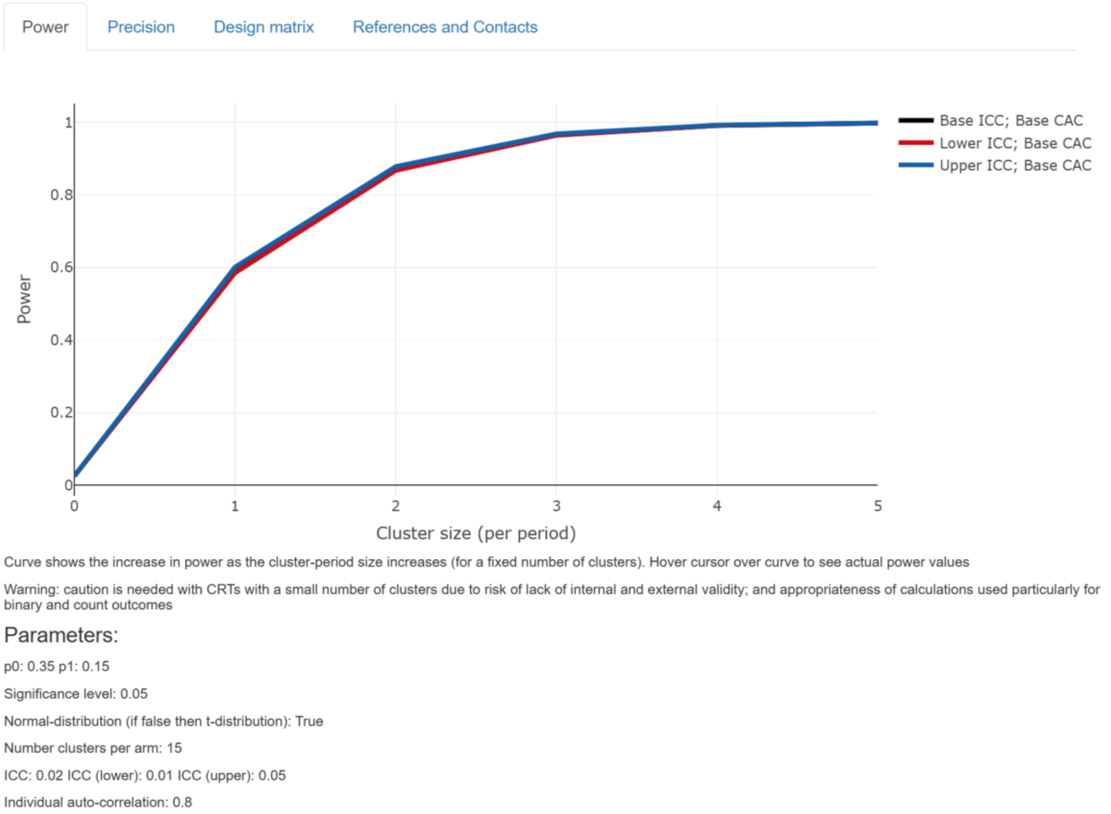

Table 1. Community health centers enrolled in this trial.

Serial number	coding	Community Name	Number of visits	Inspection quantity
1	0207	Yuetan Community Health Service Center	545337	101537
2	0217	Baizhifang Community Health Service Center	404937	77669
3	0203	Xinjiekou Community Health Service Center	403811	69171
4	0209	Zhanlanlu Community Health Service Center	374908	12230
5	0213	Taoranting Community Health Service Center	317476	7308
6	0216	Guangnei Community Health Service Center	295011	12330
7	0220	Dazhalan Community Health Service Center	232631	13731
8	0212	Shichahai Community Health Service Center	224243	26892
9	0215	Niujie Community Health Service Center	197145	31724
10	020305	Yutaoyuan Community Health Service Station	192497	20593
11	0201	Xichangan Street Community Health Service Center	173031	6701
12	020302	Xisantiao Community Health Service Station	166287	17437
13	0214	Chunshu Community Health Service Center	163692	5750
14	020301	Beiliutiao Community Health Service Station	138129	22001
15	020303	Zhaodengyu Road Community Health Service Station	115708	16173
16	021201	Shibaimi Community Health Center	104859	1811
17	020304	Guanyuan Community Health Service Station	98308	8551
18	021702	Nancaiyuan Community Health Service Station	97921	16913
19	021503	Chunfeng Community Health Service Station	90009	4334
20	021604	Changchun Street Xili Community Health Service Station	89458	1099
21	021601	Huaibaishu Community Health Service Station	87079	1265
22	021703	Shuanghuaili Community Health Service Station	76030	7777
23	021704	Youanmen Community Health Service Station	73307	11460
24	021701	Baizhifang Hutong Community Health Service Station	71598	6783
25	021202	Gulou Community Health Center	67578	1430
26	021602	Xibianmen Dongli Community Health Service Station	64155	1059
27	021205	Aimin Street Community Health Service Station	57974	2226
28	021502	Nanxiange Community Health Service Station	55429	1355
29	021203	Xisibei Community Health Service Station	53796	3163
30	021603	Sanmiao Community Health Service Station	52261	1387

Table 2. ICD-10 code for defining diseases.

Diagnosis	ICD Code Version	ICD-10 Code
Type 2 Diabetes	WHO	E11
	China	E11.900
	Beijing	E11.901
Hypertension	WHO	I10, I11, I12, I13, I15
	China	I12.900x003, I13.900, I10.x00x002, I12.900, I10.x00, I11.900, I10.x09, I15.900, I11.901
	Beijing	I13.901, I10xx11, I10xx02, I12.903, I15.901, I11.901
Cardiovascular Disease	WHO	G45, I22, I48, I63/I64, I20, J81, I11.9, I50.0, I24, I61, I11.0, I48.0, I50, I25.103, I50.1, I23, I60, I48.1, I64, I13.0, I51.6, I48.2, I25, I21
	China	I50.000, I21.900x017, I48.000, I50.904, I61.900, I50.100, I11.000, I48.100, I50.905, I50.000x006, I50.103, I50.900x018, I48.x00x015, I11.001, I48.200, I48.x01, I64.x00, I48.900x004, I50.104, I21.900, I50.105, I48.100x002, I20.900, I50.901, I50.900x007, I50.900x002, I11.002, I50.101, I48.x02, I50.902, I24.800, I13.000, J81.x00x002, I23.800, I50.102, I50.900x008, I48.x00x009, G45.900, I50.001, I50.907, I50.900x017, I25.103, I48.x00, I60.900, G45.900x001, J81.x00, I50.100x006, I50.002, I48.x00x023, I50.900, I25.900, I50.903, I48.x00x011, I50.900x009, I50.000x005, I11.900, I48.100x003, I50.908, I50.900x010, I51.600
	Beijing	I60.901, I25.105, J81xx01, I50.904, I50.905, I48xx15, I50.004, I50.103, J81xx02, I13.001, I48xx04, I11.001, I20.902, I50.106, I48xx02, I48xx08, I50.910, I50.006, I50.003, I21.902, G45.901, I50.902, I48xx13, I50.102, I48xx07, I48xx06, I50.107, I50.001, I50.907, I50.005, I50.909, I64xx02, I11.901, I64xx04, J81xx03, I50.917, I50.908, I48xx14, I48xx09, I50.911, I50.104, I48xx11
Acute Kidney Injury	WHO	N17, N01, T79.5, D59.3, K76.7, O90.4, O08.4, N99.0
	China	N10.x00, N10.x01, N00.900x009, N10.x00x003, N12.x00x005, N17.001, N00.951, N00.901, N13.801, N17.251
	Beijing	N00.908, N10xx03, N10xx04+H20.9
Obesity	WHO	E66.8, E66.2, E66.1, E66.9
	China	E23.609, E23.605, E23.600x016, E66.100x001, E66.800, E66.100, E66.801, E66.901, E66.200x001, E66.900, E66.200
	Beijing	E66.201, E23.609, E66.101, E66.801, E66.901, E23.616
Kidney stone	WHO	N20.2, N20.1, N20.0
	China	N20.901, N20.001, N20.100, N20.002, N20.900, N20.200, N20.000
	Beijing	N20.003, N20.201, N20.901, N20.101, N20.000y003, N20.005, N20.902
CKD	WHO	N18.916, N18.917, N18.915, N18.914, N18.918
	China	N18.803, N18.400, N18.200, N18.804, N18.500, N18.300, N18.801, N18.802, N18.001, N18.100
	Beijing	N18.916, N18.917, N18.915, N18.914, N18.918
Pregnancy	WHO	O10.2, O26.8, O23.2, O22.1, O30.9, O15.2, O24, O12.0, O26.6, O15.1, O22, O33.0, O26.9, O15, O33.4, O22.3, O34.1, O12.2, O34.2, O14.2, O13, O23.0, O24.2, O23.5 , O21.0, O12.1, O20.8, O26.0 , O32.4, O22.0, O14.9, O34.8, O22.9, O34.3, O20.0, O30.0, O30.8, P08.0, O26.5, O22.2, O30.1, O33.5, O21.1, O25 , O24.4, O22.4,

		O23.4, O23.3, O10.0, O21.8, O10.3, O34.7, O25, O30.2, O23, O26.4, O33.2, O34.5, O26.2, O26.1 , O21.9, O23.5, O26.7, O15.0, O22.8, O16, O34.4, O34.6, O23.1, O33.3, O14.1, O14.0, O10.1, O11, O99.0, O10.4, O20, O26.3, O22.5, O10, O21.2, O34.0, O33.1
	China	O21.000, O34.004, O21.900, O26.200, O23.400, O33.102, O21.100, O23.901, O34.801, O30.801, O24.100x011/O24.100x021, O26.607, O26.700, O20.900, O21.001, O22.800, O22.900, O30.000, O34.701, O33.500, O10.201, O34.802, O24.400, O33.400, O26.701, O24.301, O15.000, O34.003, O34.805, O30.100, O34.101, O23.001, O15.101, O22.101, O30.200, O21.200, O25.x00, O26.802, O33.200, O34.601, O13.x02, O34.600, O10.401, O22.103, O24.000, O34.804, O26.805, O26.800, O26.300, O34.603, O34.402, O22.901, O34.806, O32.400, O10.101, O34.005, O24.200x001, O14.000, O26.500, O26.900, O10.900, O10.301, O15.900, O33.100, O15.201, O22.000, O34.300, O34.602, O26.604, O33.001, O26.100, O12.100, O22.801, O26.501, O34.400, O22.500, O23.300, O26.605, O26.602, O10.200, O34.001, O34.401, O13.x01, O33.501, O21.800, O33.101, O23.500, O20.800, O30.800, O26.606, O16.x00, O26.400, O22.400, O24.900, O33.301, O23.900, O26.804, O34.200, O10.001, O33.300, O15.001, O24.100, O11.x00, O23.200, O23.000, O26.807, O10.300, O99.007, O10.400, O22.102, O34.406, O22.100, O22.300, O26.000, O34.002, O24.200, O24.300, O34.102, O34.803, O34.800, O34.500, O14.101, O34.201, O22.200, O23.505, O24.000x011/O24.000x021, O34.301, O34.502, O13.x00, O99.006, O33.201, O15.100, O26.601, O34.403, O20.000, O14.100, O23.502, O34.404, O11.x01, O26.603, O15.200, O26.801, O22.104, O14.900, O12.200, O23.504, O25.x01, O34.700, O22.902, O26.806, O23.100, O23.501, O10.000, O23.101, O26.803, O33.002, O30.900, O23.503, O10.100, O24.300x001, O26.600, O34.501, O34.604, O12.000, O34.405, O23.506, O14.102, O99.005
	Beijing	O34.004, O12.101, O34.504, O34.801, O23.901, O30.801, O34.503, O20.001, O99.003, O23.401, O15.901, O26.700, O12.201, O21.001, O26.812, O10.201, O34.802, O34.703, O34.816, O34.302, O26.701, O11xx01, O24.301, O21.903, O23.001, O15.101, O22.101, O34.601, O33.105, O26.609, O10.401, O22.103, O25xx02, O26.608, O34.603, O34.402, O20.901, O23.002, O22.901, O34.408, O15.202, O10.101, O34.005, O99.0018, O99.0017, O23.508, O10.301, O99.0016, O30.901, O34.808, O34.602, O26.604, O26.401, O22.801, O26.501, O21.201, P08.002, O24.401, O30.201, O25xx01, O26.201, O33.004, O23.201, O12.001, O26.605, O34.401, O23.511, O26.602, O34.001, O22.001, O16xx01, O33.501, O13xx01, O33.101, O24.201, O30.001, O26.101, O30.101, O33.005, O33.301, O99.207, O10.001, O15.001, O14.103, O34.819, O13xx03, O14.901, O22.102, O26.301, O34.407, O99.002, O22.100, O34.002, O34.102, O23.902, O34.803, O14.001, O34.201, O23.505, O22.903, O26.001, O22.501, O15.102, O33.201, O26.601, O22.201, O26.814, O34.403, O34.404, O23.504, O22.401, O23.101, O23.506, O14.102, O22.301
Renal Replacement Therapy	China	Z49.101
	Beijing	Z49.101

Table 3. Diagnose criteria for AKI/AKD.

AKD diagnostic criteria	Content
SCr diagnostic criteria for AKI/AKD	meet any one of the following conditions:<br>○ SCr rises ≥ 26.5 μmol/L within 48 hours<br>○ SCr decreases by ≥ 26.5 μmol/L within 48 hours<br>○ SCr rises by 50% within 7 days<br>○ SCr decreases by 50% within 7 days<br>○ SCr rises by 50% within 7 to 30 days<br>○ SCr decreases by 50% within 7 to 30 days<br>○ eGFR rises by 35% within 3 months<br>○ eGFR decreases by 35% within 3 months<br>○ The current eGFR is less than 60 ml /min/1.73m^2 and the eGFR within 3 months is greater than 60 ml /min/1.73m^2<br>○ (Extended criteria) SCr increased or decreased by > 50% compared with baseline creatinine (baseline creatinine is defined as the median creatinine within 8-365 days)
ICD -10 code for AKI/AKD	○ WHO ICD-10 code: N17, N01, T79.5, D59.3, K76.7, O90.4, O08.4, N99.0<br>○ China ICD-10 code: N10.x00, N10.x01, N00.900x009, N10.x00x003, N12.x00x005, N17.001, N00.951, N00.901, N13.801, N17.251<br>○ Beijing ICD-10 code: N00.908, N10xx03, N10xx04+H20.9
Field search for AKI/AKD	"acute renal failure", "acute kidney injury", "acute kidney disease", "acute renal failure"

AKI, acute kidney injury; AKD, acute kidney disease; SCr, serum creatinine; eGFR, estimated glomerular filtration rate.

Table 4. Safety reminder for hypoglycemic drugs.

Drug Class	Common medications	Medication Reminders from Chinese Diabetic Nephrology Clinical Prevention and Treatment Guidelines	Medication reminders
Biguanides	Metformin	○ eGFR 45-59: dose reduction.<br>○ eGFR <45: contraindication.<br>○ Accumulation may cause lactic acidosis.<br>○ Under stress (severe disturbances, acute heart failure, respiratory failure): discontinue.<br>○ Patients with diabetes mellitus (eGFR 45-60): discontinue metformin 48 hours before angiography or general anesthesia; reinitiation if without renal function deterioration after at least 48 hours.	eGFR 45-59: dose reduction<br>eGFR < 45: contraindication
Sulfonylureas	Glyburide	○ eGFR 30-59: dose reduction<br>○ eGFR < 30: contraindication	eGFR < 60: contraindication
	Glimepiride		eGFR 30-60: dose reduction<br>eGFR <30: contraindication
	Gliclazide		eGFR < 30: contraindication
	Glipizide		eGFR < 30: contraindication
	Gliquidone	○ eGFR 15-29: use with caution<br>○ eGFR < 15: contraindication	eGFR < 30: contraindication
Thiazolidinediones	Pioglitazone	○ Severe renal dysfunction: contraindication	eGFR < 30, contraindication
	Rosiglitazone	○ Decreased renal function: no dosage adjustment required	No restrictions
Meglitinides	Nateglinide	○ eGFR <15: use with caution	eGFR < 15: dose reduction
	Repaglinide	○ eGFR < 30, kidney transplantation and dialysis: reduction.	eGFR < 30: dose reduction
α-glucosidase inhibitors	Acarbose	○ eGFR < 25: contraindication	eGFR <25: dose reduction
	Voglibose	○ eGFR < 30: use with caution	eGFR <30: dose reduction
	Miglitol	/	<30, contraindication
DPP-4 inhibitors	Sitagliptin	○ eGFR 30-50: dose reduction by half<br>○ eGFR < 30: dose reduction by one-quarter	eGFR < 45: dose reduction
	Saxagliptin	○ eGFR < 45: dose reduction by half	eGFR < 45: dose reduction
	Vildagliptin	○ Moderate to severe renal insufficiency: dose reduction	eGFR < 45: dose reduction
	Linagliptin	○ No dose adjustment is required.	No restrictions
	Alogliptin	○ Moderate renal impairment: dose reduction by half<br>○ Severe renal impairment: dose reduction by 3/4	eGFR 30-60: dose reduction by half<br>eGFR <30: dose reduction by 3/4
GLP-1 receptor	Exenatide	○ eGFR > 30: no restrictions	eGFR < 30: contraindication

agonists	Liraglutide	○ ESRD: not recommended	
	Lixisenatide		
SGLT2 inhibitors	Dapagliflozin	○ eGFR <60: not recommended	eGFR < 60: contraindication
	Empagliflozin	○ eGFR <45: contraindication	eGFR < 45: contraindication
	Canagliflozin	○ eGFR 45-60: maximal dose of 100mg/day<br>○ eGFR <45: not recommended	eGFR 45-60: dose reduction<br>eGFR <45: contraindication

eGFR, estimated glomerular filtration rate; GLP-1, glucagon-like peptide-1; ESRD, end-stage renal disease; SGLT2, sodium-glucose cotransporter 2

Table 5. Details of the RE-AIM framework.

Reach	CDSS research/proportion of subjects in the total patient population
	Number of patients meeting inclusion and exclusion criteria
	Proportion of people at high risk of kidney disease who have completed kidney disease screening
	CKD patients diagnosed after CDSS intervention
Efficacy	The primary and secondary outcomes according to 4.3
Adoption	Number of general practitioners using CDSS for more than 3 and 6 months
Implementation	Number of CKD referrals
	The utilization rates of RAASi in CKD patients and SGLT2i in CKD patients
Maintenance	Community doctors’ satisfaction with the system at 3 and 6 months after the start of CDSS intervention
	Community doctors' willingness to continue using the system at 3 and 6 months after the start of CDSS intervention
	Community doctors’ subjective evaluation of whether their work efficiency has improved at 3 and 6 months after the start of CDSS intervention
	Community doctors' subjective evaluation of whether there is early warning fatigue at 3 and 6 months after the start of CDSS intervention
	Community Physicians' CKD Cognition Questionnaire at 6 months before and after the CDSS intervention

CDSS, clinical decision support systems; CKD, chronic kidney disease; RAASi, renin-angiotensin-aldosterone system; SGLT2, sodium-glucose cotransporter 2.

Table 6. CKD Cognition Questionnaire

**Questionnaire on CKD Cognition**			
Age (years)		Gender	
Experience	Job Title		
	Length of time working as a physician		
	Time spent on diagnosis and treatment of chronic diseases		
1. Are there any patients with chronic kidney disease in your clinic?		□ Yes<br>□ No	
2. Which guidelines do you usually follow to diagnose and treat CKD? (Multiple choices)		○ No guideline<br>○ KDOQI<br>○ KDIGO<br>○ Chronic Kidney Disease Screening, Diagnosis, and Prevention Guidelines<br>○ Other hypertension and diabetes guidelines as supplements	
3. Which of the following is the most common risk factor for CKD? (Single answer)		□ Age<br>□ Obesity<br>□ Hypertension<br>□ Diabetes<br>□ Cardiovascular disease<br>□ Family history of kidney disease	
4. Which of the following eGFR ranges is considered CKD stage 3? (Single answer)		□ 60-90 ml/min/1.73m^2<br>□ 30-60 ml/min/1.73m^2<br>□ 15-30 ml/min/1.73m^2<br>□ <15 ml/min/1.73m^2<br>□ I don't understand	
5. If a patient has risk factors for CKD, what tests should you arrange for the patient? (Multiple choices)		○ Serum creatinine<br>○ Serum creatinine and estimated eGFR<br>○ Urinalysis (urine protein, urine occult blood)<br>○ uACR	
6. If a patient with diabetic kidney disease has a blood pressure of 145/90 mmHg, a urine albumin/creatinine ratio of 80 mg/g, and an eGFR of 65 ml/min/1.73 m^2 measured in your clinic, and has previously taken metformin to control blood sugar.		The patient's preferred antihypertensive drug is<br>□ RAASi<br>□ CCBs<br>□ Beta-blockers<br>□ Diuretics	
		The blood pressure reduction goal for this patient is<br>□ < 140/90 mmHg<br>□ < 135/85 mmHg<br>□ < 130/80 mmHg<br>□ < 125/75 mmHg	
		Which oral hypoglycemic drug should be preferred for this patient?<br>□ SGLT2i	

	□ Acarbose<br>□ Repaglinide
7. Which of the following drugs should be used with caution in patients with kidney disease? (Multiple choices)	○ Ibuprofen, Motrin<br>○ RAASi<br>○ Rabeprazole, pantoprazole<br>○ Nifedipine
8. What are the common complications in CKD patients? (Multiple choices)	○ Anemia<br>○ Hyperkalemia<br>○ Metabolic bone disease<br>○ Cardiovascular disease
9. Which of the following situations require referral for CKD patients? (Multiple choices)	○ Anemia<br>○ Hyperkalemia<br>○ Metabolic bone disease<br>○ Cardiovascular disease<br>○ CKD stage 4~5 patients

KDOQI, Kidney Disease Outcomes Quality Initiative; KDIGO, Kidney Disease: Improving Global Outcomes; CKD, chronic kidney disease; eGFR, estimated glomerular filtration rate; uACR, urine albumin-creatinine ratio; RAASi, renin-angiotensin aldosterone inhibitors; CCB, calcium channel blockers.
