# Supplementary material for: Clinical Decision Support for Chronic Kidney Disease in Primary Care: A Cluster Randomized Clinical Trial
Source: JAMA Netw Open. 2026 May 8;9(5):e2611112. doi: 10.1001/jamanetworkopen.2026.11112 (PMC13156789; doi:10.1001/jamanetworkopen.2026.11112)
Supplement: Supplement 2. — eFigure 1. Study Design eFigure 2. Historical Trends in Process Measures and Clinical Outcomes at Participating Study Sites (2018–2022) Prior to CDSS Intervention. eTable 1. Characteristics of Participating Centers and Primary Care Physicians eTable 2. Historical Trends in Process Measures and Clinical Outcomes at Participating Study Sites (2018–2022) Prior to CDSS Intervention. [file jamanetwopen-e2611112-s002.pdf]

Supplemental Online Content

Zheng X, Hui M, Yang H, et al. Clinical decision support for chronic kidney disease in primary care: a cluster randomized clinical trial. *JAMA Netw Open*. 2026;9(5):e2611112. doi:10.1001/jamanetworkopen.2026.11112

eFigure 1. Study Design
eFigure 2. Historical Trends in Process Measures and Clinical Outcomes at Participating Study Sites (2018–2022) Prior to CDSS Intervention.
eTable 1. Characteristics of Participating Centers and Primary Care Physicians
eTable 2. Historical Trends in Process Measures and Clinical Outcomes at Participating Study Sites (2018–2022) Prior to CDSS Intervention.

This supplemental material has been provided by the authors to give readers additional information about their work.

© 2026 Zheng X et al. *JAMA Network Open*.

eFigure 1. Study Design

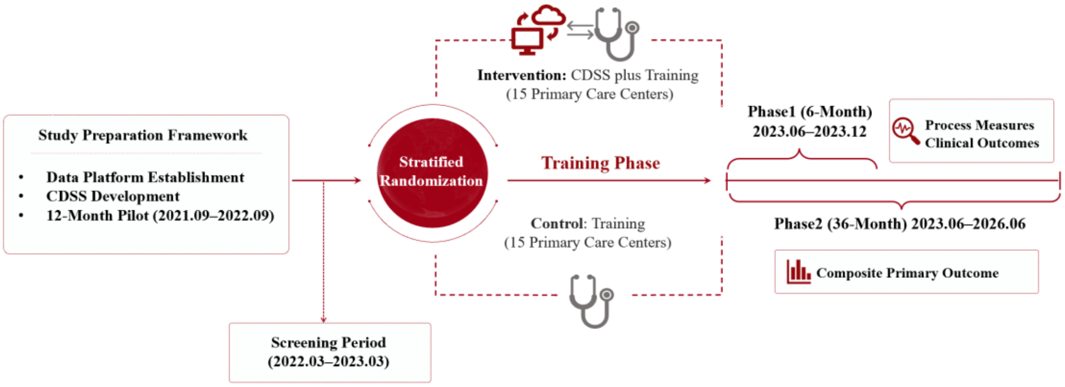

EHR, electric health record; CDSS, clinical decision support system; CKD, chronic kidney disease

eFigure 2. Historical Trends in Process Measures and Clinical Outcomes at Participating Study Sites (2018–2022) Prior to CDSS Intervention.

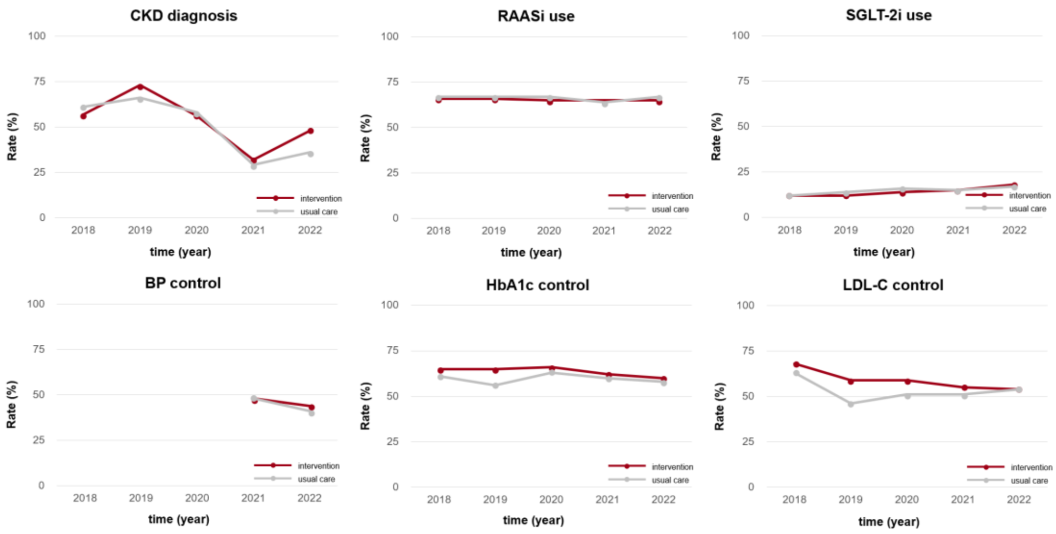

Abbreviations: CKD, chronic kidney disease; RAASi, renin-angiotensin-aldosterone system inhibitors; SGLT-2i, sodium-glucose cotransporter 2 inhibitors; BP, blood pressure; HbA1c, hemoglobin A1C; LDL-C, low-density lipoprotein cholesterol.

© 2026 Zheng X et al. *JAMA Network Open*.

eTable 1. Characteristics of Participating Centers and Primary Care Physicians

	Total	Intervention	Control
**Centers**	N=30	n=15	n=15
**Center size**			
Large	10	5	5
Medium	12	6	6
Small	8	4	4
**Annual patient visits**			
Median per center (IQR)	5,358 (3,500–20,119)	6,856 (4,506–20,538)	3,914 (2,896–18,283)
Total number	318,454	174,674	143,780
**CKD patients included**			
Median per center (IQR)	35 (14-190)	65 (21-217)	22 (12-146)
Total number	3,390	1,912	1,478
**CKD diagnosis rate at baseline, % (n/N)**	54.9 (1,862/3,390)	59.8 (1,143/1,912)	48.6 (719/1,478)
By center size:			
Large	55.2 (1,462/2,650)	62.1 (860/1,385)	47.6 (602/1,265)
Medium	51.9 (308/593)	53.3 (235/441)	48.0 (73/152)
Small	61.7 (92/149)	55.2 (48/87)	71.0 (44/62)
ACR testing available, n (%)	7 (23.3)	3 (20.0)	4 (26.7)
**Primary care physicians**	**N=428**	**n=219**	**n=186**
Number of PCPs per center, Median (IQR)	6 (4-19)	6 (5-20)	7 (4-19)
By center size:			
Large	28 (19–42)	42 (20–43)	23 (19–38)
Medium	6.5 (5–7)	6 (5–7)	7 (6–8)
Small	5 (4–5)	4 (3–5)	5 (4–5)
PCP background, n (%)			
Bachelor's degree or above	253 (85.2)	128 (88.3)	125 (82.2)
>5 years of experience	289 (97.3)	139 (95.9)	150 (98.7)

© 2026 Zheng X et al. *JAMA Network Open*.

eTable 2. Historical Trends in Process Measures and Clinical Outcomes at Participating Study Sites (2018–2022) Prior to CDSS Intervention.

	Intervention centers<br>Patients, No./total No. (%)					Usual care centers<br>Patients, No./total No. (%)				
Year	2018	2019	2020	2021	2022	2018	2019	2020	2021	2022
CKD patients, n	2733	6456	7872	4125	4841	2134	5307	6313	4208	5457
CKD diagnosis, n (%)	1554/2733 (56.8%)	4708/6456 (72.9%)	4437/7872 (56.4%)	1305/4125 (31.6%)	2344/4841 (48.4%)	1307/2134 (61.2%)	3512/5307 (66.2%)	3630/6313 (57.5%)	1209/4208 (28.7%)	1955/5457 (35.8%)
RAASi use, n (%)	1814/2733 (66.4%)	4244/6456 (65.7%)	5155/7872 (65.5%)	2662/4125 (64.5%)	3154/4841 (65.2%)	1423/2134 (66.7%)	3532/5307 (66.6%)	4254/6313 (67.4%)	2697/4208 (64.1%)	3635/5457 (66.6%)
SGLT-2i use, n (%)	315/2733 (11.5%)	801/6456 (12.4%)	1108/7872 (14.1%)	631/4125 (15.3%)	869/4841 (18.0%)	255/2134 (11.9%)	736/5307 (13.9%)	990/6313 (15.7%)	652/4208 (15.5%)	910/5457 (16.7%)
BP control, n (%)	NA	NA	NA	272/562 (48.4%)	1749/3998 (43.7%)	NA	NA	NA	239/497 (48.1%)	1722/4207 (40.9%)
HbA1c control, n (%)	299/458 (65.3%)	631/975 (64.7%)	1402/2136 (65.6%)	559/907 (61.6%)	490/813 (60.3%)	160/261 (61.3%)	286/509 (56.2%)	697/1102 (63.2%)	433/725 (59.7%)	419/721 (58.1%)
LDL-C control, n (%)	526/779 (67.5%)	1058/1789 (59.1%)	1893/3205 (59.1%)	997/1829 (54.5%)	899/1653 (54.4%)	412/649 (63.5%)	689/1514 (45.5%)	1339/2640 (50.7%)	1230/2390 (51.5%)	1493/2788 (53.6%)

Abbreviations: CKD, chronic kidney disease; RAASi, renin-angiotensin-aldosterone system inhibitors; SGLT-2i, sodium-glucose cotransporter 2 inhibitors; BP, blood pressure; HbA1c, hemoglobin A1C; LDL-C, low-density lipoprotein cholesterol.

© 2026 Zheng X et al. *JAMA Network Open*.
