# Supplementary material for: Clinical Decision Support for Chronic Kidney Disease in Primary Care: A Cluster Randomized Clinical Trial
Source: JAMA Netw Open. 2026 May 8;9(5):e2611112. doi: 10.1001/jamanetworkopen.2026.11112 (PMC13156789; doi:10.1001/jamanetworkopen.2026.11112)
Supplement: Supplement 3. — Data Sharing Statement [file jamanetwopen-e2611112-s003.pdf]

Data Sharing Statement

Zheng. Clinical Decision Support for Chronic Kidney Disease in Primary Care. *JAMA Netw Open*. Published May 08, 2026. doi:10.1001/jamanetworkopen.2026.11112

Data
Additional Information: ChiCTR2300070555
Data available: No
